# A new species of *Egglestonichthys* (Teleostei, Gobiiformes, Gobiidae) from Okinawa Island, Japan

**DOI:** 10.3897/zookeys.1006.58874

**Published:** 2020-12-21

**Authors:** Kyoji Fujiwara, Toshiyuki Suzuki, Hiroyuki Motomura

**Affiliations:** 1 The United Graduate School of Agricultural Sciences, Kagoshima University, 1-21-24 Korimoto, Kagoshima 890-0065, Japan Kagoshima University Kagoshima Japan; 2 Osaka Museum of Natural History, 1-23 Nagai Park, Higashi-sumiyoshi, Osaka 546-0034, Japan Osaka Museum of Natural History Osaka Japan; 3 The Kagoshima University Museum, 1-21-30 Korimoto, Kagoshima 890-0065, Japan The Kagoshima University Museum Korimoto Japan

**Keywords:** Deepwater, description, morphology, Ryukyu Islands, taxonomy

## Abstract

*Egglestonichthys fulmen***sp. nov.** (Teleostei: Gobiidae) is described on the basis of a single specimen (21.7 mm in standard length) collected from 250 m depth off Okinawa Island, Ryukyu Islands, Japan. The new species is characterized by the following combination of characters: anal-fin rays I, 9; pectoral-fin rays 17, lower rays not free from membrane; longitudinal scale series 25; transverse scales 8; pre-dorsal-fin scale rows 8; cheek and opercle naked; pelvic frenum absent; caudal fin lanceolate, its length 32.2% of SL; interorbital width very narrow, 1.2% of HL (much narrower than pupil diameter); no spicules or odontoid processes on outer surface of gill arches; and body whitish, upper half with broken zigzag pattern of bright yellow patches and associated scattered black melanophores in fresh specimens (melanophores retained in preserved specimens). Several characters, including pectoral-fin ray count, interorbital width, and coloration uniquely distinguish the new species from congeners.

## Introduction

On 19 September 2018, an unidentified goby was collected by basket trap from 250 m depth off Okinawa Island, Japan, and subsequently identified as a species of *Egglestonichthys* Miller & Wongrat, 1979. Although a generic reassessment of the genus *Egglestonichthys* is needed (Larson 2013), it currently includes five valid species [*Egglestonichthys bombylios* Larson & Hoese, 1997, *Egglestonichthys melanoptera* (Visweswara Rao, 1971), *Egglestonichthys patriciae* Miller & Wongrat, 1979, *Egglestonichthys rubidus* Allen, Erdmann & Brooks, 2020, and *Egglestonichthys ulbubunitj* Larson, 2013] (Larson 2013; Allen et al. 2020). Although additional Japanese specimens could not be collected due to the difficulty of collecting small, benthic fishes from deeper coastal areas, the present specimen was quite distinct from other congeners in meristics, morphometrics, and coloration, and is here formally described as a new species.

## Materials and methods

Counts and measurements follow Larson (2013). Measurements were made to the nearest 0.01 mm, except for standard length (nearest 0.1 mm), with needle-point calipers under a dissecting microscope. Standard and head lengths are abbreviated as SL and HL, respectively. Cephalic sensory papillae and head and body squamation were observed using versatile staining with Cyanine Blue (Saruwatari et al. 1997). Osteological elements were examined from radiographs. OMNH is the institutional code for Osaka Museum of Natural History, Osaka, Japan. Comparative data for species of *Egglestonichthys* were taken from Larson and Hoese (1997) [*E. bombylios* and *E. melanoptera*], Winterbottom and Burridge (1992) [*E. patriciae*], Larson (2013) [*E. ulbubunitj*], and Allen et al. (2020) [*E. rubidus*].

## Results

### Egglestonichthys fulmen sp. nov.

Taxon classificationAnimaliaPerciformesGobiidae

88B739F5-42C5-5430-9038-7E279D6BA746

http://zoobank.org/52DCCC67-7462-446E-A2DA-078489CE92D5

**Figure 1.**
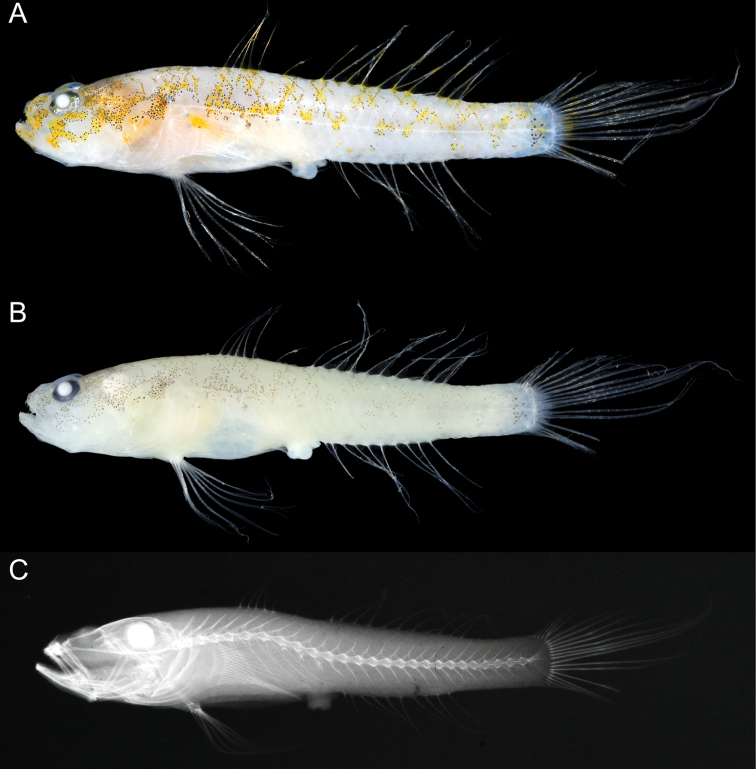
Holotype of *Egglestonichthys fulmen* sp. nov., OMNH-P 43993, 21.7 mm SL, Okinawa Island, Ryukyu Islands, Japan **A** fresh coloration**B** preserved coloration**C** radiograph.

**Figure 2.**
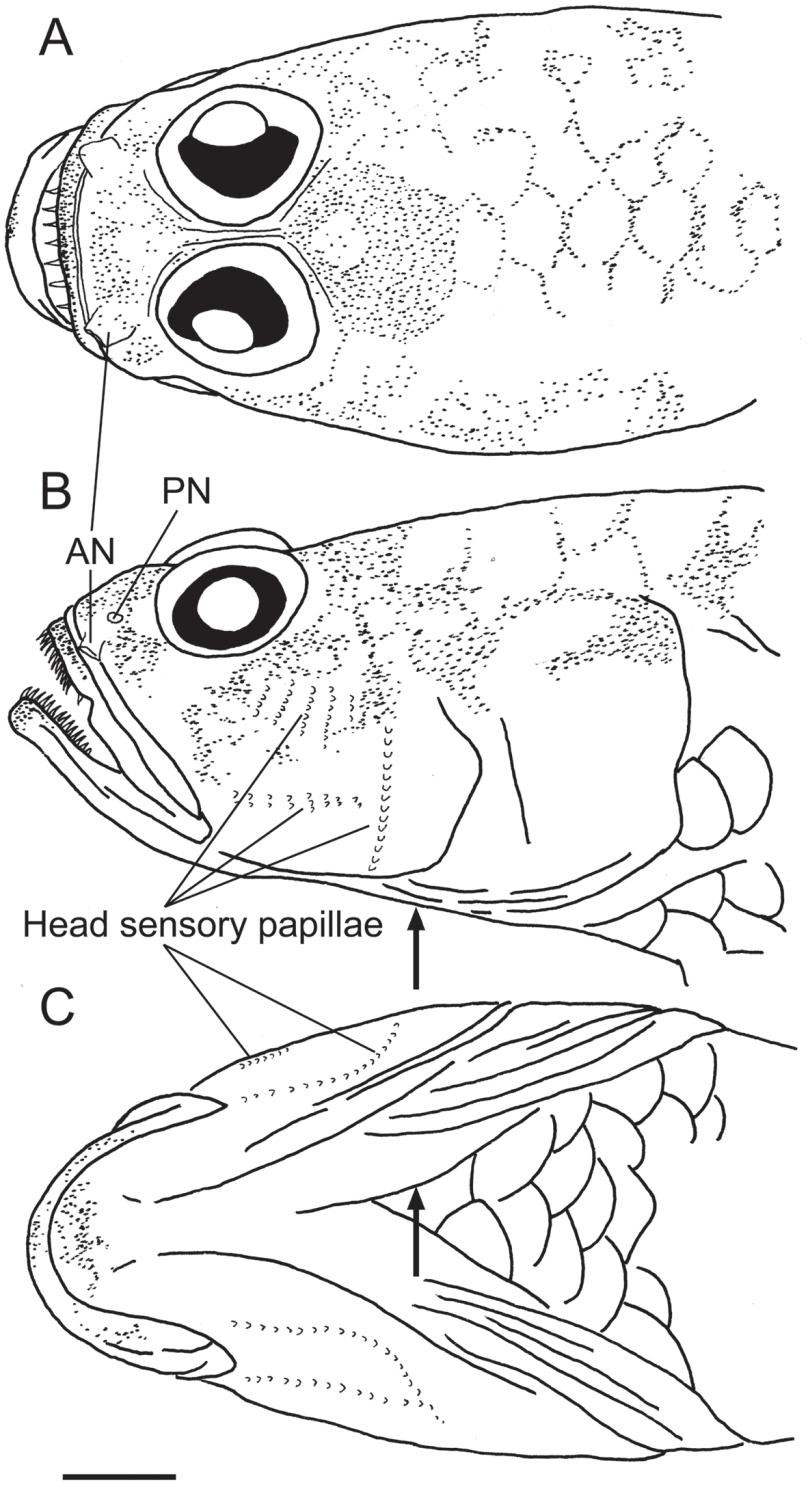
Sketches of head region of *Egglestonichthys fulmen* sp. nov. based on OMNH-P 43993, holotype, 21.7 mm SL. Note that sensory papillae have been abraded during capture **A** dorsal view **B** lateral view **C** ventral view. AN and PN indicate anterior and posterior nostrils, respectively. *Arrowheads* indicate anteroventral end of gill opening. Scale bar: 1 mm.

Figures 1 , 2 New English name: Eggleston’s Lightning Goby New standard Japanese name: Raitei-haze

#### Holotype.

OMNH-P 43993, 21.7 mm SL, Hamahiga Island, off Okinawa Island, Ryukyu Islands, Japan, 250 m depth, 19 Sept. 2018, basket trap, K. Abe.

#### Diagnosis.

A species of *Egglestonichthys* (Fig. 1) with the following combination of characters: anal-fin rays I, 9; pectoral-fin rays 17, lower rays connected by membrane; longitudinal series scales 25; transverse scales 8; pre-dorsal-fin scale rows 8; cheek and opercle naked (Fig. 2B); pelvic frenum absent; caudal fin lanceolate, its length 32.2% of SL; interorbital width very narrow, 1.2% of HL (much narrower than pupil diameter) (Fig. 2A); no spicules or odontoid processes on outer surface of gill arches; and body whitish, upper half with broken zigzag pattern of bright yellow patches and associated scattered black melanophores in fresh specimens (melanophores retained in preserved specimens) (Fig. 1A, B).

#### Description.

Dorsal-fin rays VI + I, 10; anal-fin rays I, 9; pectoral-fin rays 17; segmented caudal-fin rays 16; caudal-fin ray pattern 9/7; branched caudal-fin rays 7/5; unsegmented (procurrent) caudal-fin rays 6/6; longitudinal series scales 25; transverse scales 8; pre-dorsal-fin scale rows 8 (counted from scale pockets); circumpeduncular scales 12; gill rakers on outer face of first arch 3 + 11 (counted on right side); vertebrae 10 + 16. The following morphometrics are expressed as percentage of SL (% of HL in parentheses): head length 30.3; head depth 16.7 (55.1); head width 18.3 (60.6); body depth 14.9; body width 12.9; caudal-peduncle length 19.1, depth 9.4; snout length 5.9 (19.3); eye diameter 7.3 (24.0); interorbital width 0.4 (1.2); upper-jaw length 11.8 (39.0); pectoral-fin length 31.0; pelvic-fin length 28.1; caudal-fin length 32.2; and longest dorsal-fin spine (2^nd^) length 12.8.

Body slender, compressed, width much less than depth (Fig. 1A, B). Anus situated just before anal-fin origin. Head short, depressed anteriorly, its width slightly greater than height. Snout short (slightly shorter than eye diameter), rounded, slightly inflated. Anterior and posterior nostrils close to each other; former located just above anterior upper lip, much larger than latter, with long membranous tube; latter located between snout tip and anterior margin of eye, small, circular. Eye moderate, located dorsolaterally. Interorbital region very narrow, flattened. Mouth terminal, anteriorly inclined obliquely upwards. Lower jaw longer than upper jaw, its posterior tip reaching back to vertical through middle of pupil. Upper-jaw tip behind vertical through lower-jaw tip. Both jaws with two or three irregular rows of small, pointed conical teeth, the tip of each slightly curved posteriorly. Gill opening wide, anteroventral point extending slightly forward to vertical level of preopercle margin, gill membranes attached anteriorly to isthmus. Preopercular and opercular margins slightly pointed and rounded, respectively, upper end of latter horizontally level with middle of eye. Gill rakers sharp, slender. Outer surfaces of gill arches lacking spicules or odontoid processes.

Head lacking sensory canal pores (Fig. 2). Head sensory papillae (many damaged by abrasion and unobservable) in transverse pattern on cheek (possibly more complex in undamaged condition); two rows of longitudinal papillae under head (Fig. 2).

Body covered with relatively large cycloid scales. Pre-dorsal- and pelvic-fin regions covered with cycloid scales; anterior margin of pre-dorsal-fin scales reaching to vertical through preopercle margin; pre-pelvic-fin scales reaching to just behind anteroventral point of gill opening. Pectoral-fin base with 2 relatively large cycloid scales. Side of head (snout, suborbital region, jaws, cheek, and opercle) naked (Fig. 2).

First dorsal fin triangular, 2^nd^ spine longest, all spines lacking filamentous tips; dorsal-fin origin located posterior to vertical through pectoral-fin base; all spines thin, flexible. Second dorsal and anal fins long, origin of latter under base of 1^st^ dorsal-fin soft ray, anterior 1–3 rays of both fins somewhat short, thereafter subequal in length (longer than dorsal-fin spines), last rays well separated from caudal-fin base. Pectoral fin long, pointed, middle rays longest, tips reaching to below origin of 2^nd^ dorsal-fin soft ray; all rays connected by membrane, uppermost and lower 3 rays unbranched. Pelvic fins completely connected by membrane, without frenum; posterior tip reaching below 2^nd^ dorsal-fin origin when appressed; pelvic-fin origin located just below ventral end of pectoral-fin base. Caudal fin lanceolate, its length much greater than head length.

#### Coloration.

***Fresh coloration*** (Fig. 1A). Body whitish, the upper half with a broken zigzag pattern of bright yellow patches, extending from below 1^st^ dorsal-fin origin to caudal-fin base. Head ground color whitish with bright yellow markings comprising three and two short, poorly defined bars and stripes, respectively, on snout and under eye; 1^st^ and 2^nd^ bars on snout, 3^rd^ bar connecting with two stripes under eye; indistinct grayish or yellowish markings on upper part of head (behind eye, upper part of opercle, and pre-dorsal region). Scattered black melanophores throughout bright yellow patches on head and body. Dorsal-fin rays whitish with yellow interspaces (possibly barred in undamaged specimens). Other fins whitish; upper end of pectoral-fin base with yellow spot; caudal-fin base with faint yellow vertical bar and broad rectangular patch of melanophores.

***Coloration when preserved*** (Fig. 1B). Head and body uniformly whitish. Black melanophores (associated with yellow patches when fresh) retained.

#### Distribution.

Currently recorded only from Okinawa Island, Ryukyu Islands, Japan, from a depth of 250 m.

#### Etymology.

The specific name “*fulmen*” is derived from Latin, meaning “lightning”, in reference to the bright yellow zigzag pattern on the upper part of the body.

#### Remarks.

The holotype and only known example of the new species lacks head sensory pores, but has a transverse sensory papillae pattern on the cheek and a wide gill opening (the anteroventral point extending slightly forward to be vertically level with the preopercle margin), thereby matching the diagnostic characters of *Egglestonichthys* given by Miller and Wongrat (1979). According to Winterbottom and Burridge (1992), *Egglestonichthys* is the sister-group of *Priolepis* Valenciennes, both genera sharing the above characters. Except for a few species (e.g., *Priolepis goldshmidtae* Goren & Baranes and *Priolepis winterbottomi* Nogawa & Endo; Fujiwara et al. 2020), *Priolepis* has spicules or odontoid processes on the outer surface of the 1^st^ gill arch and vertical dark-margined bars on the head and body, and these are regarded as generic diagnostic characters (Winterbottom and Burridge 1992). Although Larson and Hoese (1996) pointed out that similar characters were also present in some species of *Egglestonichthys* (e.g., spicules present in *E. melanoptera* and *E. patriciae*; head and body with dark brown bands in *E. bombylios* and *E. patriciae*), neither could be confirmed in the present specimen. A detailed investigation of species currently assigned to *Egglestonichthys* must precede any redefinition of the genus (see Larson 2013: 153). Nevertheless, in addition to the above-mentioned characters, the present specimen is here considered much closer to *Egglestonichthys* than to *Priolepis* in general appearance (particularly in the lanceolate caudal fin, which is not found in any species of *Priolepis*).

*Egglestonichthys fulmen* is unlikely to be misidentified as one of its congeners, having the following characters: 17 pectoral-fin rays (vs. 20–22 in *E. bombylios* and *E. melanoptera*; 20 in *E. patriciae*; and 19–21 in *E. ulbubunitj*); interorbit very narrow (width 1.2% of HL), much less than pupil diameter [vs. variously broad: 38.5–47.6% HL in *E. bombylios* (described as 4.9–5.9 in HL in Larson and Hoese 1996); 17.0–20.4% HL in *E. melanoptera* (4.9–5.9 in HL in Larson and Hoese 1996); equal to pupil diameter in *E. patriciae* (proportion not provided in Winterbottom and Burridge 1992); 14.9–17.0% HL in *E. rubidus* (4.3–5.4 in SL in Allen et al. 2020), and 14.0–23.2% HL in *E. ulbubunitj*]; and body whitish, the upper part with a broken zigzag pattern of bright yellow patches in fresh specimens, and associated black melanophores in both fresh and preserved specimens (vs. body pale to brownish with dark brown bands or blotches in fresh and preserved specimens of *E. bombylios*, *E. patriciae*, and *E. ulbubunitj*, generally uniformly dark brown to black in *E. melanoptera*, and generally uniformly red and dark brown in fresh and preserved conditions, respectively, in *E. rubidus*).

Although *E. fulmen* and *E. melanoptera* both lack a pelvic frenum, the former has much lower scale counts than the latter (25 longitudinal series scales, 8 transverse scales, and 8 pre-dorsal-fin scale rows in *E. fulmen* vs. 35–45, 12–14, and 29–37, respectively, in *E. melanoptera*). Larson (2013) described the lower 3–7 pectoral-fin rays being free of membrane as a unique character of *E. ulbubunitj*; all are connected by membrane in *E. fulmen*. In addition, *E. fulmen* has fewer anal-fin rays (I, 9 vs. I, 10 in *E. ulbubunitj*). *Egglestonichthys fulmen* also has fewer longitudinal series scales than *E. bombylios* and *E. patriciae* (25 vs. 31–35 and ca. 40, respectively), a lanceolate caudal fin (length 32.2% of SL), and scales absent on the cheek and opercle (vs. caudal fin truncated, and cheek and opercle with ctenoid scales in both of the latter).

## Supplementary Material

XML Treatment for Egglestonichthys fulmen
